# Junctophilin-2 tethers T-tubules and recruits functional L-type calcium channels to lipid rafts in adult cardiomyocytes

**DOI:** 10.1093/cvr/cvaa033

**Published:** 2020-02-13

**Authors:** Claire Poulet, Jose Sanchez-Alonso, Pamela Swiatlowska, Florence Mouy, Carla Lucarelli, Anita Alvarez-Laviada, Polina Gross, Cesare Terracciano, Steven Houser, Julia Gorelik

**Affiliations:** 1 National Heart and Lung Institute, Imperial College London, Du Cane Road, London W12 0NN, UK; 2 Department of Cardiac Surgery, School of Medicine, University of Verona, Piazzale L.A. Scuro 10, 37134 Verona, Italy; 3 Cardiovascular Research Center, Lewis Katz School of Medicine, Temple University, 3500 N. Broad St., Philadelphia, PA 19140, USA

**Keywords:** Junctophilin-2, T-tubules, L-type calcium channels, Cholesterol, Lipid rafts, Caveolin3

## Abstract

**Aim:**

In cardiomyocytes, transverse tubules (T-tubules) associate with the sarcoplasmic reticulum (SR), forming junctional membrane complexes (JMCs) where L-type calcium channels (LTCCs) are juxtaposed to Ryanodine receptors (RyR). Junctophilin-2 (JPH2) supports the assembly of JMCs by tethering T-tubules to the SR membrane. T-tubule remodelling in cardiac diseases is associated with downregulation of JPH2 expression suggesting that JPH2 plays a crucial role in T-tubule stability. Furthermore, increasing evidence indicate that JPH2 might additionally act as a modulator of calcium signalling by directly regulating RyR and LTCCs. This study aimed at determining whether JPH2 overexpression restores normal T-tubule structure and LTCC function in cultured cardiomyocytes.

**Methods and results:**

Rat ventricular myocytes kept in culture for 4 days showed extensive T-tubule remodelling with impaired JPH2 localization and relocation of the scaffolding protein Caveolin3 (Cav3) from the T-tubules to the outer membrane. Overexpression of JPH2 restored T-tubule structure and Cav3 relocation. Depletion of membrane cholesterol by chronic treatment with methyl-β-cyclodextrin (MβCD) countered the stabilizing effect of JPH2 overexpression on T-tubules and Cav3. Super-resolution scanning patch-clamp showed that JPH2 overexpression greatly increased the number of functional LTCCs at the plasma membrane. Treatment with MβCD reduced LTCC open probability and activity. Proximity ligation assays showed that MβCD did not affect JPH2 interaction with RyR and the pore-forming LTCC subunit Ca_v_1.2, but strongly impaired JPH2 association with Cav3 and the accessory LTCC subunit Ca_v_β2.

**Conclusions:**

JPH2 promotes T-tubule structural stability and recruits functional LTCCs to the membrane, most likely by directly binding to the channel. Cholesterol is involved in the binding of JPH2 to T-tubules as well as in the modulation of LTCC activity. We propose a model where cholesterol and Cav3 support the assembly of lipid rafts which provide an anchor for JPH2 to form JMCs and a platform for signalling complexes to regulate LTCC activity.

## Graphical Abstract

**Figure:**
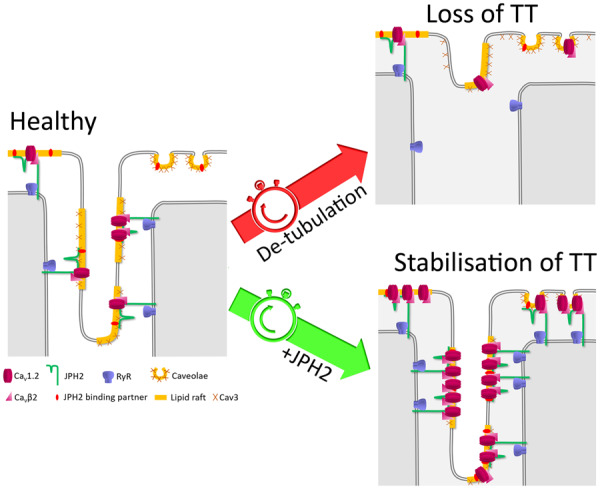

## 1. Introduction

The plasma membrane of adult cardiomyocytes is organized in a regular network of invaginations called transverse tubules or T-tubules (reviewed in reference 1). The T-tubule network consists of transverse elements, which run to the centre of the cells along the Z-line of sarcomeres; and longitudinal extensions. T-tubules associate with the sarcoplasmic reticulum (SR), forming junctional membrane complexes (JMCs) where L-type calcium channels (LTCCs) are juxtaposed to Ryanodine receptors (RyR) of the SR (reviewed in reference 2). JMCs, which, to a lesser extent, also occur underneath the surface membrane as peripheral couplings, are essential for the translation of action potentials into muscle contraction, a process called excitation–contraction coupling (EC coupling)^1^: when a wave of depolarization reaches a cell, it propagates throughout the cell along the T-tubules and activates the opening of LTCCs. A small influx of Ca^2+^ through LTCCs triggers a much larger Ca^2+^ release from the SR via the opening of RyR, a phenomenon known as Ca^2+^-induced Ca^2+^ release (CICR). Calcium ions subsequently bind to the nearby sarcomeres, inducing their contraction. Subsequent removal of cytosolic Ca^2+^ via the SR Ca^2+^-ATPase SERCA, as well as the Na^+^/Ca^2+^ exchanger and the plasma membrane Ca^2+^-ATPase, both highly concentrated in the T-tubules, allow for rapid relaxation of the cell.

Structural deterioration of T-tubules has been repeatedly observed in animal and human cardiomyopathies. Alterations include reduced regularity in the distribution of transverse tubules together with increased proportion of longitudinal elements^3–8^; loss of T-tubule openings at the membrane^8^^,^^9^; and reduced number and length of JMCs.^10^^,^^11^ Areas of extensive T-tubule remodelling show delayed and lower Ca^2+^ release due to impaired CICR.^3^^,^^4^^,^^8^^,^^12–14^ This results in a general dyssynchrony of Ca^2+^ release through the cells which participates to the pathophysiology of failing myocytes. The severity of T-tubule remodelling increases with the development of heart failure^4^^,^^5^ and correlates with cardiac contractile dysfunction.^5^ Interestingly, disruption of the tubular network starts early during the progression of the disease, before any effect on cardiac function can be noted.^5^ Hence, it has been suggested that T-tubule remodelling might play a critical role in the development of heart failure.

While the mechanisms underlying T-tubule disruption are yet to be fully elucidated, evidence suggest that the structural protein junctophilin-2 (JPH2) is crucial for the maintenance of T-tubule integrity (reviewed in reference 2). JPH2 was first identified by Takeshima *et al.*^15^ as the protein forming JMCs in cardiomyocytes. With a C-terminal transmembrane segment that spans the SR membrane and a repetition of conserved MORN motifs in the N-terminal region which interacts with the plasma membrane, JPH2 forms a bridge between T-tubules and the SR, maintaining a distance of ∼12 nm between the two membranes.^15^

Animal models of JPH2 knockdown show reduced number and length of JMCs,^15^^,^^16^ loss of T-tubule regularity,^16^ as well as impaired T-tubule maturation during postnatal development.^17^^,^^18^ The consequences of these alterations are irregular and smaller calcium transients, cardiac contractile dysfunction, heart failure, and increased mortality.^16^^,^^19^ Silencing JPH2 in cultured myocytes similarly affects T-tubules structure and calcium transients.^5^^,^^10^

JPH2 expression is down-regulated in animal and human cardiomyopathies,^5^^,^^6^^,^^11^^,^^20–25^ and its localization altered: while JPH2 is distributed in a regular striated pattern in healthy myocytes, the protein forms aggregates in failing cells,^7^ leaving areas devoid of JPH2 which most likely correlate with areas of extensive T-tubule remodelling. Interestingly, restoring JPH2 expression by indirect methods improved T-tubule structure in several disease models.^6^^,^^7^^,^^24^^,^^26^^,^^27^ Taken together, these results suggest that downregulation of JPH2 might be a common mechanism underlying the disruption of T-tubules in cardiomyopathies of different aetiology.

The nature of the interaction between JPH2 and the plasma membrane is unclear. It has been suggested that JPH2 associates with the scaffolding protein Caveolin3 (Cav3),^20^ which, together with cholesterol, supports the formation of small flask-shaped membrane invaginations called caveolae. Caveolae are a subset of lipid rafts, membrane microdomains of ordered lipids and proteins that play a role in many cellular processes such as signal transduction. While the presence of caveolae in T-tubules remains controversial,^28^^,^^29^ it has been repeatedly shown that Cav3 lines the T-tubular membrane^29^^,^^30^ where it could act as a binding partner for JPH2.

In addition to its structural role in JMCs, JPH2 seems to have regulatory functions on calcium signalling. Within JMCs, JPH2 and RyR associate and form large clusters.^31^ Increasing evidence indicate that JPH2 modulates the activity of RyR by stabilizing the open probability of the channel.^16^^,^^32^^,^^33^ Furthermore, immunoprecipitation assays identified a potential association between JPH2 and the LTCC pore-forming subunit Ca_v_1.2.^25^^,^^34^ The nature of this interaction is unclear but experiments in a mammalian expression system showed that Ca_v_1.2 activity was altered in the presence of JPH2.^25^ These results suggest that JPH2 might regulate LTCCs, but whether this is true in cardiomyocytes remains to be determined.

The present study examined the role of JPH2 in T-tubule stability and LTCC regulation in cardiomyocytes. We monitored T-tubule remodelling in rat ventricular myocytes placed in primary culture and found that JPH2 overexpression prevented the disruption and eventual loss of T-tubules that occur in these conditions. Interestingly, the maintenance of T-tubular structures by JPH2 overexpression depended on the presence of cholesterol in the plasma membrane. Using super-resolution scanning patch clamp, we also demonstrated that JPH2 recruited functional LTCCs to the plasma membrane and that cholesterol was involved in the modulation of LTCC activity. These new data support a model where JPH2 directly interacts with both Ca_v_1.2 and RyR to facilitate CICR, and where cholesterol and Cav3 support the assembly of lipid rafts in T-tubules, which provide an anchor for JPH2 to form JMCs to house signalling complexes for the regulation of LTCCs.

## 2. Methods

### 2.1 Study approval

All animal experiments were carried out in accordance with the United Kingdom Home Office Animals (Scientific Procedures) Act 1986 Amendment Regulations 2012, incorporating the EU Directive 2010/63/EU, which conforms to the Guide for the Care and Use of Laboratory Animals published by the US National Institutes of Health (NIH publication No. 85-23, revised 1996).

### 2.2 Chemical and drugs

Unless otherwise stated, all chemical and drugs were purchased from Sigma-Aldrich.

### 2.3 Cardiomyocyte isolation and culture

Ventricular myocytes were isolated from the hearts of male adult Sprague-Dawley rats (150–250 g) as described in the Supplementary material online. The animals were anaesthetized with 5% isoflurane-95% O_2_ and killed by cervical dislocation. Myocytes were plated on laminin-coated coverslips or glass-bottom dishes (MatTek) in modified M199 (Invitrogen, UK) culture medium containing bovine serum albumin (0.5 g/L), creatine (5 mmol/L), taurine (5 mmol/L), L-ascorbic acid (100 µmol/L), carnitine (2 mmol/L), and penicillin/streptomycin (100 mmol/L). For JPH2 overexpression, myocytes were infected with Ad-h-JPH2 adenovirus (Vector BioLabs) on the day of isolation with a multiplicity of infection of ∼200 virus particles per cell. For chronic treatment with cyclodextrin, 100 µmol/L Methyl-β-cyclodextrin was added to the cells every day.

### 2.4 Antibodies

For immunostaining, proximity ligation assay and western blot, the following antibodies were used: anti-JPH2 (sc-51313, Santa Cruz Biotechnology), anti-Cav3 (610421, BD Biosciences) anti-RyR2 (HPA020028, Sigma-Aldrich), anti-Ca_v_1.2 (ACC-013, Alomone Labs), and anti-Ca_v_β2 (kindly provided by Doctor Ravi Balijepalli).

### 2.5 T-tubule staining, immunostaining, and image analysis

Protocols for immunostaining and staining of T-tubules with Di-8-ANEPPS are given in the Supplementary material online. Density and power of regularity were measured as previously described.^35^ Briefly, an area of 5 × 40 µm was selected in each image and binarized. Density was calculated as the fraction of black pixels. The binarized images were plotted as waveforms which were converted by Fast Fourier Transform into a representation of the frequency of recurring signals using a custom-written macro for Matlab. As the distance between two T-tubules is ∼2 µm, power values were measured at a frequency of ∼0.5 cycles per micron.

### 2.6 Proximity ligation assay

Myocytes on coverslips were fixed with ice-cold methanol for 5 min at −20°C and blocked with 10% foetal bovine serum in PBS for 30 min at room temperature. Incubation with primary antibodies was done in blocking buffer overnight at 4°C. Proximity ligation assay was then performed using the Duolink system (Sigma-Aldrich). Images were taken with a Zeiss LSM-780 inverted confocal microscope. Staining density was calculated by normalizing the area covered by the signal to the area of the cell.

### 2.7 SICM and super-resolution scanning patch clamp

Scanning ion conductance microscopy (SICM) was used to generated high resolution 3D images of the surface of live cells using nanopipettes as scanning probes.^36^ Super-resolution scanning patch clamp to record single LTCC activity was performed at regions of T-tubule openings or in crest areas as previously described^37^ and as detailed in the Supplementary material online. Channel density was determined by normalizing the total number of channels recorded to the sum of pipette tip inner diameters which were calculated using the following equation: *R*_pipette_ = (*π* · *d*/2 · tan(*ϕ*/2) · *p*)^−^^1^, where *R*_pipette_ is the pipette resistance, *d* the pipette tip inner diameter, *ϕ* the tip cone angle (∼3.8°), and *p* the conductivity of the pipette solution (∼1.8 S/m).^38^

### 2.8 Whole cell patch-clamp

Whole cell Ca^2+^ currents were measured using the conventional voltage-clamp technique in the ruptured patch configuration as described in the Supplementary material online.

### 2.9 Statistical analysis

Data are shown as scatter/dot plots with means ± SEM. Statistical comparison was done using unpaired Student’s *t*-test or one-way analysis of variance (ANOVA) followed by Tukey’s multiple comparison test to compare mean values among all conditions, or Bonferroni’s test to compare selected sets of means, as defined in the figure legends.

## 3. Results

### 3.1 JPH2 is rapidly down-regulated and disorganized in culture

**Figure 1:**
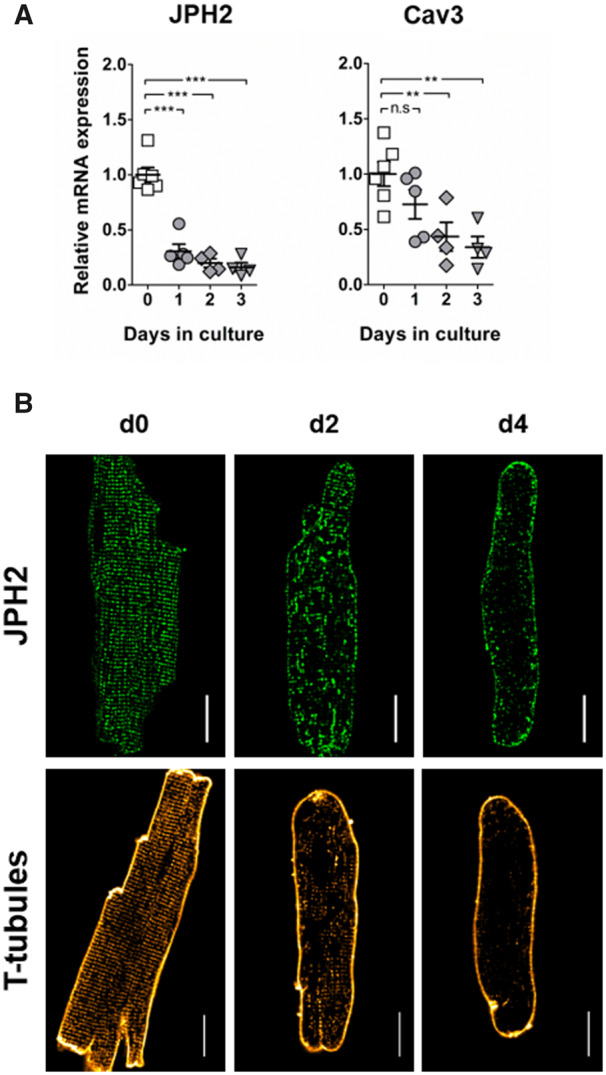
JPH2 expression is rapidly down-regulated in culture and its disorganization accompanies the remodelling of T-tubules. (*A*) Gene expression of JPH2 and Cav3 at different times of culture. The dots represent the number of isolations: *n* = 6 (Day 0); *n* = 5 (Day 1); *n* = 4 (Day 2); *n* = 4 (Day 3). (*B*) Immunostaining for JPH2 and membrane staining with Di-8-ANEPPs to show T-tubules in freshly isolated myocytes, and after 2 (d2) and 4 (d4) days of culture. Scale bar: 20 µm.

We measured the gene expression of JPH2 and Cav3 in cultured cardiomyocytes (*Figure 1A*) and found that JPH2 was significantly down-regulated after only 24 h with a reduction of 70% compared to freshly isolated cells. In contrast, 2 days of culture were needed to see a reduction of Cav3 expression to 55%.

In fresh cardiomyocytes (d0), JPH2 was organized in a very regular, punctuated pattern (*Figure 1B*). After 2 days of culture however, its distribution was extensively remodelled. The protein formed large aggregates inside the cells or relocalized to the membrane, leaving areas completely devoid of JPH2. After 4 days of culture most JPH2 was found at the membrane. This disorganization of JPH2 accompanied the remodelling of T-tubules that has been previously reported in cultured cardiomyocytes.^12^^,^^39^ The very regular striated pattern of the T-tubule network in freshly isolated cardiomyocytes was indeed progressively remodelled in culture, until complete loss after 4 days. As expected we observed alterations in cell morphology due to culture conditions. While many cells ‘collapsed’ to a more spherical shape (Supplementary material online, *Figure S1A*), a fraction of myocytes retained an elongated shape, though more elliptical than freshly isolated cells as cell ends became rounded. Image analysis and patch clamp experiments were performed only on such elongated cells. We found a reduction of ∼25% of cell volume in these cells after 4 days of culture (Supplementary material online, *Figure S1B*).

### 3.2 JPH2 overexpression prevents the remodelling of JPH2, Cav3, and T-tubules

**Figure 2:**
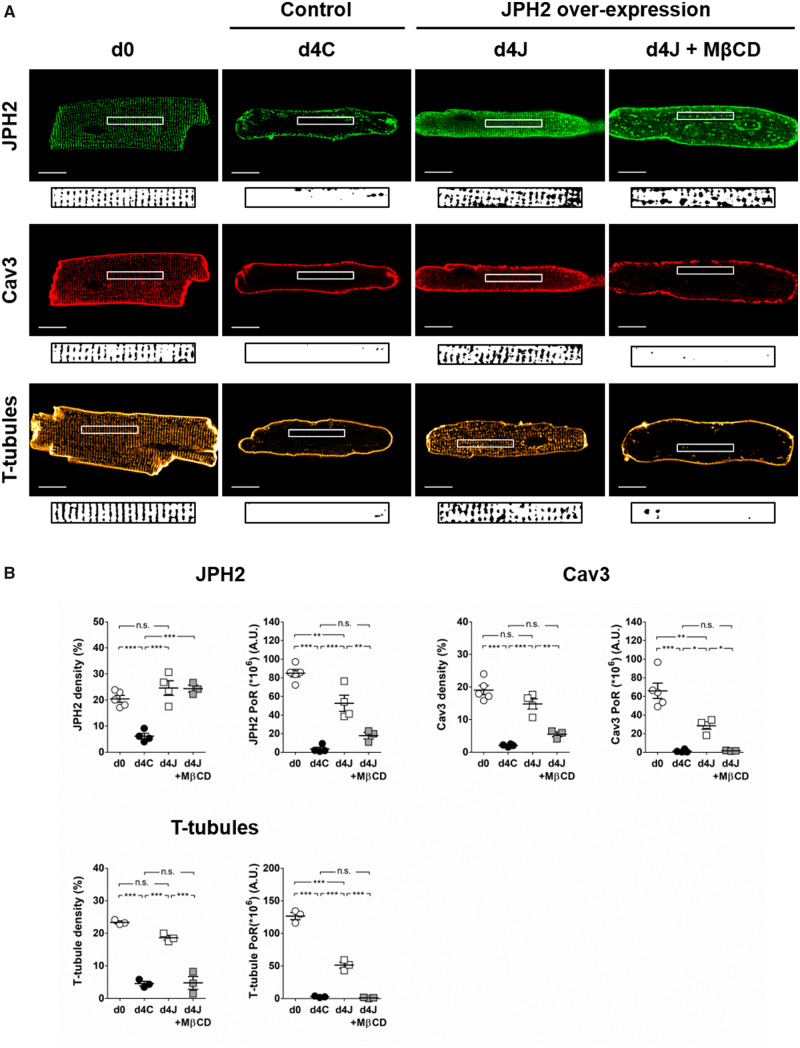
Overexpression of JPH2 prevents the loss of Cav3 and T-tubules in culture via cholesterol domains. (*A*) Distribution of JPH2, Cav3, and T-tubules in freshly isolated cardiomyocytes (d0) and after 4 days of culture in control cells (d4C) or in cells overexpressing JPH2 in basal conditions (d4J) or chronically treated with 100 µmol/L MβCD (d4J + MβCD). Scale bar: 20 µm. An area of 40 × 5µm was selected in each cell (white rectangle) and binarized as shown under each picture to calculate staining density and power of regularity shown in (*B*). Average values were calculated from five cells per isolation. The dots represent the number of isolations. For JPH2 and Cav3 stainings: *n* = 5 (d0); *n* = 4 (d4C); *n* = 3 (d4J); *n* = 3 (d4J + MβCD). For T-tubule staining: *n* = 3 for each condition. *P < 0.05, ** *P* < 0.01, *** *P* < 0.0001, one-way ANOVA followed by Tukey’s multiple comparison test.

JPH2 overexpression in cultured myocytes was confirmed at both transcript and protein levels (Supplementary material online, *Figure S2*). We found that JPH2 overexpression prevented its own relocalization as the protein exhibited a striated organization even after 4 days of culture (d4J, *Figure 2A*). Density and regularity of JPH2 staining (*Figure 2B*) were strongly reduced in control cells after 4 days of culture (d4C). Myocytes overexpressing JPH2 showed significantly higher staining density and regularity than non-transfected controls. Strikingly, myocytes overexpressing JPH2 showed a striated distribution of Cav3 and a regular T-tubule network while these structures were not observed in non-transfected controls. These observations were confirmed by the quantification of Cav3 and T-tubule staining density and regularity which were significantly higher in cells overexpressing JPH2 (*Figure 2B*). The density of JPH2, Cav3 and T-tubule staining was comparable to what we observed in freshly isolated myocytes. The power of regularity, however, did not reach the levels found in d0 controls, suggesting that cells overexpressing JPH2 still underwent a moderate remodelling.

To further characterize the distribution of proteins and T-tubules through cell volume, we repeated the measurement of staining regularity in three different areas of maximum intensity projections of z-stacks (Supplementary material online, *Figure S3*). We found similar results as described above. Distribution of JPH2, Cav3 and T-tubule through cell volume is shown in movies of exemplary Z-stacks (Supplementary material online).

We validated our results using a different adenovirus construct in feline ventricular myocytes (Supplementary material online, *Figure S4*).

Interestingly, while Cav3 gene expression was strongly reduced in culture, its protein levels remained unchanged (Supplementary material online, *Figure S2*).

### 3.3 Cholesterol removal counteracts the stabilizing effect of JPH2 overexpression on T-tubules

Cultured myocytes were chronically treated with 100 µmol/L of the cholesterol sequestering drug methyl-β-cyclodextrin (MβCD) to remove cholesterol from the membrane and disrupt lipid rafts, including caveolae.^40^ Such treatment prevented the stabilizing effect of JPH2 overexpression in cultured myocytes. In myocytes overexpressing JPH2 and treated with MβCD (d4J + MβCD) the density of JPH2 staining was not changed (*Figure 2*). Its regularity was however reduced due to the formation of big clusters in the cells. Nevertheless, the distribution of JPH2 seemed to maintain some degree of organization (*Figure 2A*). In those cells, Cav3 was only seen at the plasma membrane. Density and regularity of Cav3 and T-tubule stainings were reduced to values comparable to what we observed in d4C myocytes (*Figure 2B*).

**Figure 3:**
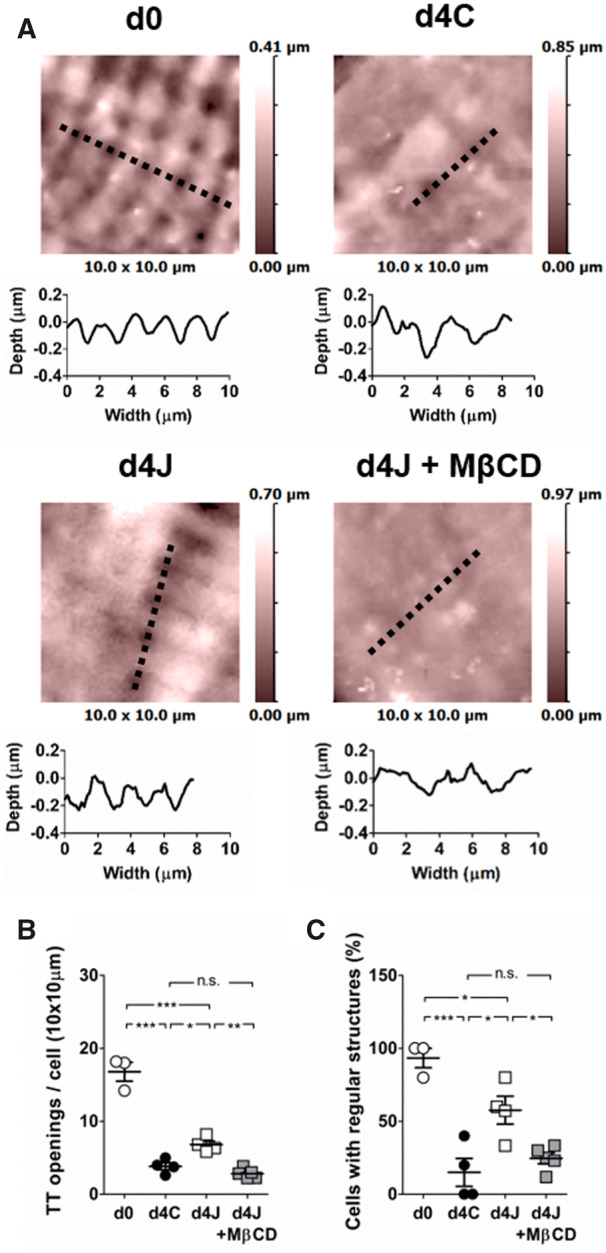
JPH2 overexpression partially stabilizes T-tubule openings at the membrane. (*A*) Examples of 3D scans recorded using SICM in freshly isolated cardiomyocytes (d0), after 4 days of culture in control cells (d4C) or in cells overexpressing JPH2 in basal conditions (d4J) or chronically treated with µmol/L MβCD (d4J + MβCD). The topographic profiles corresponding to the dashed lines are shown below each scan. (*B*) The number of T-tubules was counted in a 100 µm^2^ area for each cell (10 × 10 µm scan). (*C*) Number of cells with regular structures defined as at least four aligned openings. Average values were calculated from at least five cells per isolation. The dots represent the number of isolations: *n* = 3 (d0); *n* = 4 (d4C); *n* = 4 (d4J); *n* = 5 (d4J + MβCD). *P < 0.05, **P < 0.01, ***P < 0.0001, one-way ANOVA followed by Tukey’s multiple comparison test.

The presence of T-tubule openings at the cell surface was assessed using scanning ion conductance microscopy (*Figure 3*). Freshly isolated myocytes showed many T-tubule openings, 16.8 ± 1.3 per 100 µm^2^ that were organized in very regular rows. The number of observed T-tubule openings was drastically reduced after 4 days of culture to ∼3.8 ± 0.5 per 100 µm^2^ (*Figure 3B*), and only a few cells showed aligned structures (*Figure 3C*). In cells overexpressing JPH2, the number and regularity of openings were significantly higher than in control cells, 6.8 ± 0.5 per 100 µm^2^. When cholesterol was depleted from the membrane, the number of T-tubule openings was significantly reduced to 2.8 ± 0.3 openings per 100 µm^2^ and their alignment strongly altered.

### 3.4 JPH2 overexpression increases the number of functional LTCCs

**Figure 4:**
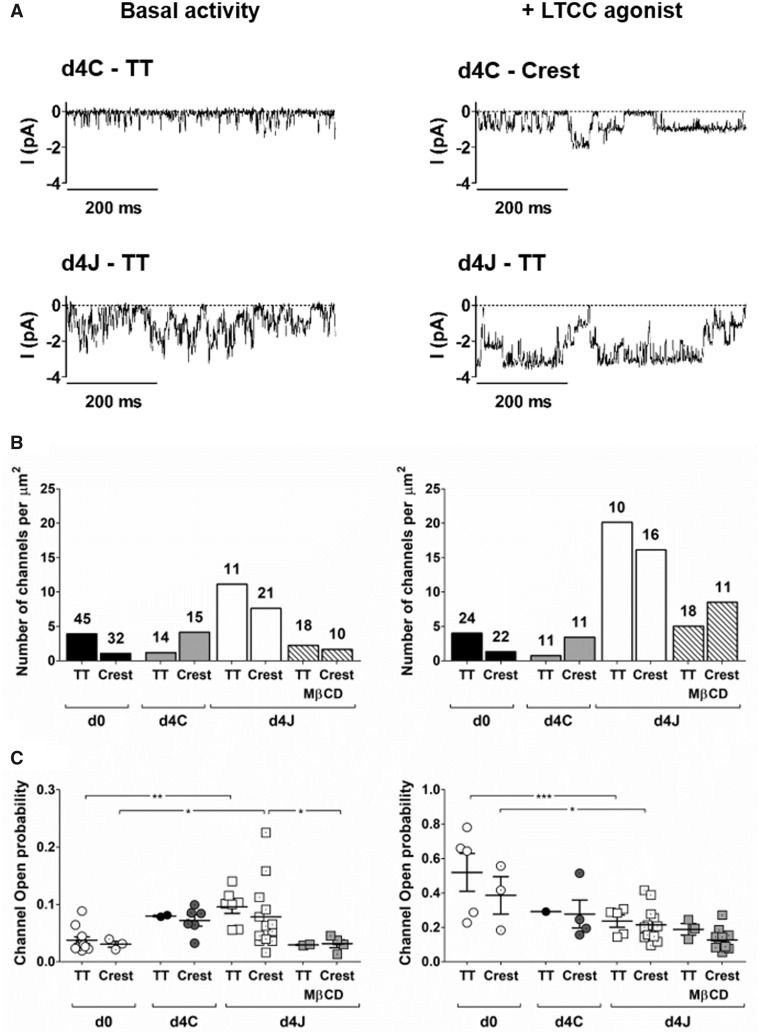
JPH2 overexpression recruits functional LTCC at the membrane. Single LTCC activity was recorded in T-tubules (TT) or non-tubular areas of the membrane (Crest) using super-resolution scanning patch clamp in basal conditions (left) or in the presence of the LTCC agonist Bay K8644 (5 µM in the pipette; right). (*A*) Examples of original recordings. From a holding potential of −96.7 mV, channels were activated by a step to −6.7 mV. (*B*) Channel density calculated per µm^2^. The number indicated for each column is the total number of patches. (*C*) Channel open probability at −6.7 mV. The dots represent the number of patches with one or more Ca^2+^ channel(s). Left graph (basal activity): *n* = 12 (d0—TT); *n* = 3 (d0—CREST); *n* = 2 (d4C—TT); *n* = 6 (d4C—CREST); *n* = 7 (d4J—TT); *n* = 13 (d4J—CREST); *n* = 2 (d4J + MβCD—TT) *n* = 4 (d4J + MβCD—CREST). Right graph (+ LTCC agonist): *n* = 5 (d0—TT); *n* = 3 (d0—CREST); *n* = 1 (d4C—TT); *n* = 4 (d4C—CREST); *n* = 5 (d4J—TT); *n* = 13 (d4J—CREST); *n* = 3 (d4J + MβCD—TT) *n* = 11 (d4J + MβCD—CREST). Notice the change of scale between left and right. *P < 0.05, **P < 0.01, one-way ANOVA followed by Bonferroni’s *post hoc* test.

We used super resolution patch-clamp to look for LTCC activity in specific microdomains of the membrane (*Figure 4*). As shown in our previous work,^37^ freshly isolated myocytes had a higher density of LTCCs in T-tubules than in non-tubular areas, or crest, under basal conditions: 3.9 vs. 1.1 channels per µm^2^, respectively (*Figure 4B*, left). After 4 days of culture, this ratio was inverted as we found 1.2 channels per µm^2^ in T-tubules and 4.1 in the crest. In cells overexpressing JPH2, LTCC density was much higher in both T-tubules and crest. Similarly to fresh myocytes, more channels were observed in T-tubules than in the crest: 11.1 vs. 7.6 channels per µm^2^, respectively. LTCC open probability was significantly higher in cells overexpressing JPH2 compared to freshly isolated myocytes (*Figure 4C*, left). As a similar, although non-significant increase was observed in non-transfected controls, this change was unlikely caused by JPH2 but rather resulted from culture time.

The increase in LTCC density in cells overexpressing JPH2 could be either due to higher channel activity or to an increase in the total number of channels present at the membrane. In order to answer that question, we repeated the experiment using the LTCC agonist Bay K8644 to induce maximal opening of all LTCCs and uncover potential elusive channels with a low open probability that could be miss in basal conditions (*Figure 4*, right).

In d0 and d4C myocytes, we did not see any change in channel density in the T-tubules or in the crest in the presence of Bay K8644, meaning that increasing the open probability with the agonist did not uncover any low activity channel. In cells overexpressing JPH2 however, the use of Bay K8644 resulted in a 2-fold increase in channel density with 20.1 and 16.1 channels per µm^2^ in T-tubules and the crest, respectively. These results corroborated that the higher LTCC activity in cells overexpressing JPH2 was due to a higher number of channels at the membrane, a fraction of which remains in an almost inactive state under basal conditions. As expected, Bay K8644 increased channel open probability in all groups. As noted above, the difference in open probability between freshly isolated myocytes and cells overexpressing JPH2 was likely due to culture conditions (*Figure 4C*, right).

**Figure 5:**
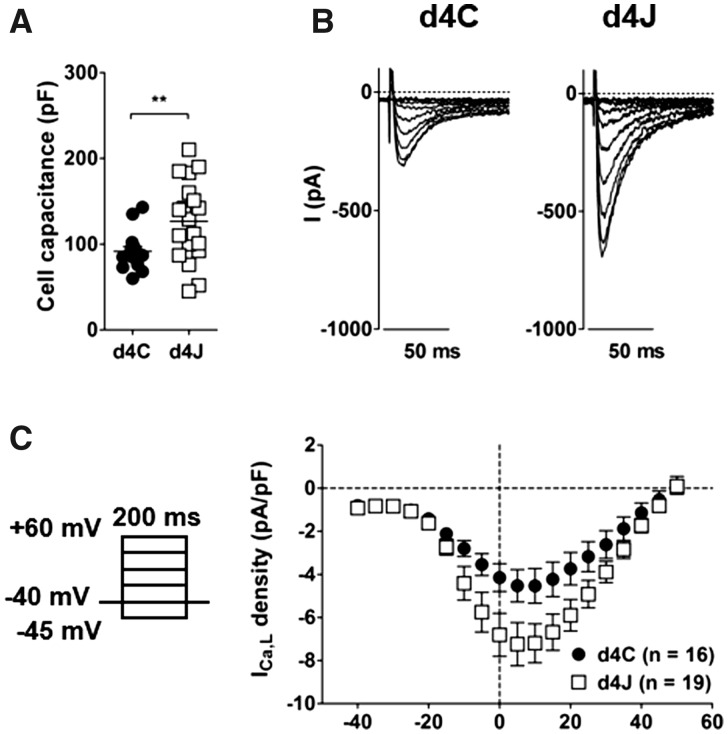
Cell capacitance and whole cell L-type Ca^2+^ current are increased in myocytes overexpressing JPH2. (*A*) Cell capacitance in control cells (d4C, *n* = 16) and cells overexpressing JPH2 (d4J, *n* = 19) after 4 days of culture. **P < 0.01, unpaired Student’s *t*-test. (*B*) Examples of whole cell L-type Ca^2+^ currents recorded at various voltages. (*C*) Left: protocol for current activation. Right: current–voltage relationships. Current at +10 mV: −4.53 ± 0.80 pA/pF in d4C and −7.20 ± 0.90 pA/pF in d4J, *P* < 0.05, Student’s *t*-test.

Using whole cell patch-clamp, we confirmed that the capacitance of cells overexpressing JPH2 was significantly higher than non-transduced controls due to the presence of T-tubules: 91.5 ± 5.4 pF in d4C controls (*n* = 16) vs. 126.7 ± 10.7 pF in d4J cells (*n* = 19), *P* < 0.01 (*Figure 5A*). We also confirmed that overexpressing JPH2 resulted in a significant increase of whole cell Ca^2+^ current, *I*_Ca,__L_: −4.53 ± 0.80 pA/pF in d4C cells (*n* = 16) and −7.20 ± 0.90 pA/pF in d4J myocytes (*n* = 19), at +10 mV, *P* < 0.05 (*Figure 5B,C*).

However, JPH2 overexpression did not prevent the reduction in Ca_v_1.2 gene and protein expression observed in culture (Supplementary material online, *Figure S2*). Therefore, the increase in LTCC density observed in cells overexpressing JPH2 is better explained by alterations in channel trafficking within the cells rather than by increased synthesis of Ca_v_1.2.

### 3.5 Cholesterol removal alters LTCC activity

When cells overexpressing JPH2 were treated with MβCD, channel density was reduced in both T-tubules and crest to 2.2 and 1.7 channels per µm^2^, respectively. In the presence of Bay K8644, channel density was twice as high in T-tubules with 5.1 channels per µm^2^, an increase that is comparable to what we observed in d4J cells non-treated with MβCD. Strikingly, Bay K8644 caused a 5-fold increase in channel density in the crest, from 1.7 to 8.5 channels per µm^2^, implying that an increased proportion of channels could not be recorded in the crest after cholesterol removal without an agonist. Furthermore, we found that MβCD reduced channel open probability in both membrane domains (*Figure 4C*). These results suggest that LTCC activity is altered by the disruption of lipid rafts.

**Figure 6:**
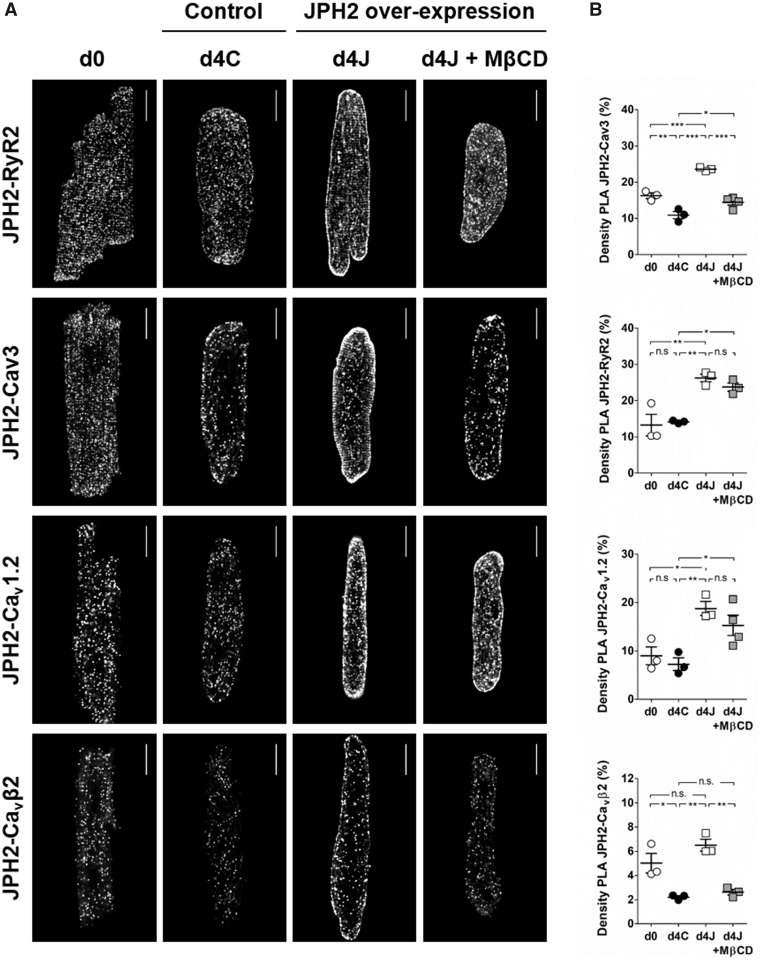
Culture time and cholesterol removal alter JPH2-Cav3 and JPH2-Ca_v_β2 interactions but do not affect JPH2-RyR2 and JPH2-Ca_v_1.2 associations. Proximity ligation assay (PLA) to detect interactions between JPH2 and RyR2, Cav3, Ca_v_1.2, or Ca_v_β2. (*A*) Examples of PLA in freshly isolated cardiomyocytes (d0) and after 4 days of culture in control cells (d4C) or in cells overexpressing JPH2 in basal conditions (d4J) or chronically treated with µmol/L MβCD (d4J + MβCD). Scale bar: 20 µm. (*B*) Staining density measured as the fraction of area covered by the signal. Average values were calculated from at least five cells per isolation. The dots represent the number of isolations. For JPH2-RyR2 and JPH2- Ca_v_β2, *n* = 3 for each condition. For JPH2-Cav3 and JPH2-Ca_v_1.2, *n* = 3 for the first three conditions, *n* = 4 for d4J + MβCD. *P < 0.05, **P < 0.01, ***P < 0.0001, one-way ANOVA followed by Tukey’s multiple comparison test.

Using proximity ligation assay, we assessed the interaction of JPH2 with RyR, Cav3, Ca_v_1.2, and Ca_v_β2, pore-forming and accessory subunits of cardiac LTCCs, respectively (*Figure 6*). We found that culture time did not influence the interaction of JPH2 with RyR and Ca_v_1.2 but reduced the association of JPH2 with Cav3 and Ca_v_β2. Overexpressing JPH2 increased all protein interactions tested. Following cholesterol removal with MβCD, we observed a strong reduction of JPH2-Cav3 and JPH2-Ca_v_β2 interactions to values found in d4C controls but the association of JPH2 with RyR and Ca_v_1.2 remained unchanged, suggesting that only the interaction of JPH2 with Cav3 and Ca_v_β2 depended on the integrity of lipid rafts.

## 4. Discussion

### 4.1 Lipid rafts are required for the binding of JPH2 to T-tubules

We found that T-tubule remodelling in cultured myocytes was associated with a very rapid reduction of JPH2 expression. This supports the idea that JPH2 downregulation is the common cause of T-tubule disruption.^6^^,^^7^^,^^24^^,^^26^^,^^27^ Moreover, we showed that overexpressing JPH2 in cultured myocytes restored T-tubule structure. Since the expression of a component of the T-tubular membrane, Cav3, was not increased in cells overexpressing JPH2 we conclude that JPH2 overexpression supported the maintenance of existing T-tubules rather than inducing the formation of new T-tubules. These results further support the idea that JPH2 plays a crucial role in T-tubule stability and that maintaining JPH2 expression might have beneficial effects on pathological cardiac remodelling. Using a mouse model of cardiac-specific JPH2 overexpression, Guo *et al.*^41^ previously reported that JPH2 overexpression, while having no effect on the cardiac performance of healthy animals, improved cardiac function under pressure overload conditions as animals overexpressing JPH2 were less disposed to develop heart failure and showed higher survival rates following transaortic constriction. This was most likely due to the increase in length and number of JMCs which helped preserve the integrity of the T-tubule network. In a more recent study, Reynolds *et al.*^42^ intravenously used adenoviral constructs to express JPH2 in mice with moderate pressure overload-induced heart failure. They found that restoring JPH2 levels prevented T-tubule remodelling, partially normalized calcium handling and, importantly, preserved cardiac function. This notable work showed that enhancing JPH2 expression using a gene-therapy approach after the onset of the disease prevented the progression of heart failure, thereby demonstrating that JPH2 could be a therapeutic target for the treatment of cardiomyopathies associated with T-tubule deterioration.

While it is clear that JPH2 tethers T-tubules to the SR, little is known about the nature of the interaction between JPH2 and the T-tubular membrane. Here, we found that depleting membrane cholesterol countered the stabilizing effect of JPH2 overexpression on T-tubule structure in cultured myocytes. Similarly, a study showed severe T-tubule remodelling in mouse cardiomyocytes following acute or chronic treatment with MβCD.^43^ Replenishing cholesterol restored T-tubule structure in the acute model and prevented further damages in cells that had been chronically treated for 24 h. Taken together these results suggest that cholesterol plays a critical role in the maintenance of T-tubule structural integrity, possibly by providing an anchor for JPH2. While cholesterol can directly bind to a panel of membrane proteins, a protein lipid assay failed to reveal any direct interaction with JPH2.^44^ These assays however showed that JPH2 interacts with other lipids such as the phospholipid phosphatidylserine. Phosphatidylserine is normally located on the cytoplasmic side of the plasma membrane but migrate to the outer side of the membrane in stressed and apoptotic cells, such as ischemic cardiomyocytes.^45^ It has been suggested that this migration might disrupt the binding of JPH2 to T-tubules and cause the remodelling of T-tubules observed in cardiovascular diseases.^44^ Other phospholipids that associate with JPH2 include various phosphoinositides (PIs), such as phosphatidylinositol 3 phosphate (PIP_3_).^44^ This interaction possibly occurs via the MORN motifs of JPH2 N-terminal domain as it was previously shown that MORN motifs in plant PI-kinases are responsible for the interaction with PIs.^46^ This agrees with the work by Takeshima *et al.*^15^ which showed that MORN motifs contribute to the binding capacity of the major skeletal muscle junctophilin, JPH1, to the plasma membrane. Interestingly, cardiomyocytes from a mouse model of PI-3-kinase (PI3K) knockout showed severe T-tubule remodelling and JPH2 mislocalization.^47^ As PI3K is responsible for the production of PIP_3_, the authors hypothesized that the structural alterations observed were due to the disruption of JPH2 binding to the plasma membrane because of a reduced pool of PIP_3_.

Another possible way for JPH2 to bind to the plasma membrane is to interact with proteins that are associated with cholesterol such as Cav3. As mentioned before, immunoprecipitations assays demonstrated a potential interaction between JPH2 and Cav3, raising the possibility that JPH2 is anchored to the plasma membrane via Cav3.^20^ In the present study, proximity ligation assays indeed showed a close association between JPH2 and Cav3 which was impaired after 4 days of culture and upon cholesterol depletion. As Cav3 protein levels remained unchanged, we believe that Cav3 was relocalized to the surface membrane rather than degraded, possibly due to a reduction in protein turnover. Whether this relocation was the trigger for T-tubule remodelling or whether it was a result from T-tubule disruption remains however unclear, as our experiments do not enable us to clarify whether Cav3 acts as an anchoring partner for JPH2. Numerous proteins physically interact with caveolins, mostly via the following caveolin-binding motifs: φXφXXXXφ or φXXXXφXXφ, where φ is an aromatic amino acid, Trp, Phe, or Tyr, and X any amino acid.^48^ We did not identify such a binding motif in JPH2. Furthermore, Cav3 overexpression failed to protect against the detrimental effect of cholesterol depletion on T-tubules.^43^ These observations therefore do not support the idea that JPH2 uses Cav3 to bind to T-tubules.

**Figure 7:**
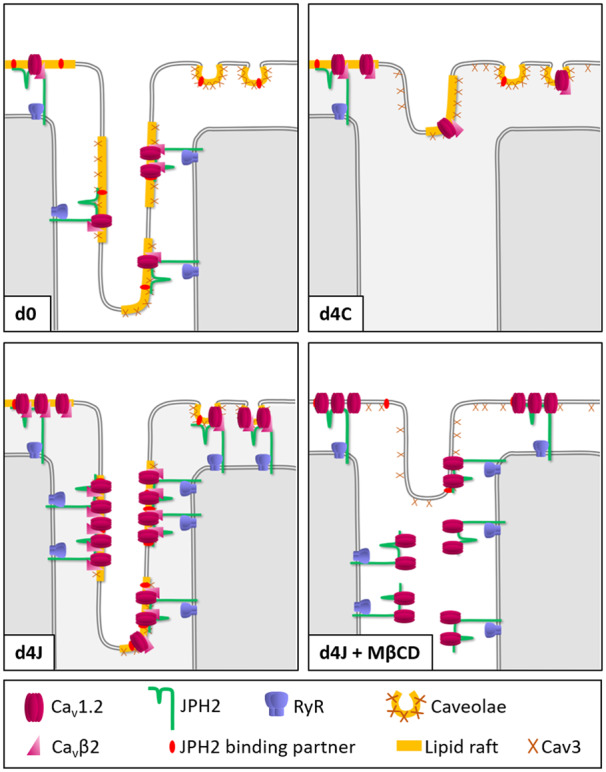
Model for the assembly of lipid rafts at JMCs and the consequences of cholesterol depletion. d0: In freshly isolated cells, more LTCCs are found in the T-tubules than in the crest. d4C: the downregulation of JPH2 in culture leads to disruption of JMCs and remodelling of T-tubules. Cav3 and LTCCs relocalize to the outer membrane and more channels are found in the crest. d4J: JPH2 overexpression prevents the remodelling of T-tubules and induces a large increase in the number of JMCs and functional LTCCs in both T-tubules and crest. d4J + MβCD: following the disruption of lipid rafts, JPH2 dissociates from the T-tubules leading to their remodelling. Cav3 relocates to the outer membrane. LTCC open probability is altered, most likely due to the dispersion of LTCC modulators such as Ca_v_β2.

Nevertheless, based on previous observations and our own results, we hypothesize that the two proteins are part of a membrane complex, whose organization is supported by cholesterol. We suggest that cholesterol forms lipid rafts in T-tubules where JPH2 and Cav3 colocalize and whose function is, amongst undoubtedly many others, to provide an anchor, lipid or protein, for JPH2. This hypothesis is further supported by the high concentration of the two major components of lipid rafts, cholesterol and sphingolipids, in T-tubules^49^; and sucrose-gradient fractionation experiments which placed JPH2 in low-density fractions where lipid rafts are usually found.^20^ Although Cav3 lines the T-tubular membrane, caveolae have been rarely observed in T-tubules, and never at sites where T-tubules form JMCs.^28^ Lipid rafts at these sites are therefore likely to be flat, while, in contrast, peripheral JMCs occur in both caveolae and flat lipid rafts.^29^ Depletion of membrane cholesterol leads to raft dissociation, explaining the detrimental effect of MβCD treatment on the tethering of T-tubules. *Figure 7* proposes a model which summarizes our hypotheses.

### 4.2 JPH2 recruits functional LTCCs

Overexpressing JPH2 in cultured myocytes increased the number of functional LTCCs in both T-tubule and sarcolemma. JPH2 overexpression did not induce the synthesis of new channels, but most likely promoted the mobilization of existing LTCCs. These results suggest that JPH2 plays a role in the recruitment of LTCCs to the plasma membrane. Our proximity ligation assays showed a strong association between JPH2 and the pore-forming α-subunit of LTCCs, Ca_v_1.2, which was not impaired by culture time or cholesterol depletion. These results support the idea that JPH2 might physically interact with the α-subunit of LTCCs, which has been suggested before in studies on skeletal muscle. Using immunoprecipitation and pull down assays, Golini *et al.*^50^ identified a region in JPH1 and JPH2 crucial for their association with the skeletal muscle LTCC α-subunit, Ca_v_1.1. They further showed that silencing JPH1 and JPH2 in cultured skeletal myotubes strongly reduced *I*_Ca,__L_ amplitude and associated this reduction to a loss of LTCCs at the plasma membrane. A very recent study identified a well conserved domain of 10 amino acids in the C-terminus of Ca_v_1.1 and Ca_v_1.2 which mediates their interaction with JPH1 and JPH2.^51^ The authors showed that silencing JPH2 impaired the colocalization of Ca_v_1.1 with RyR and reduced *I*_Ca,__L_ amplitude but did not find any evidence for a reduction in LTCC membrane expression. They concluded that JPH targets LTCCs to JMCs and enables their coupling to RyR through a physical interaction. As mentioned before, JPH2 forms clusters with RyR^31^ and modulates the activity of the channel, most likely by direct binding.^16^^,^^32^^,^^33^ Our PLA assays showed that even after JMCs were disrupted and T-tubules lost due to culture time or cholesterol depletion, JPH2 maintained its interaction with both Ca_v_1.2 and RyR. These results suggest that JPH2 supports the coupling of Ca_v_1.2 with RyR by directly interacting with both channels, a model which would greatly facilitate CICR and guarantee a highly efficient EC coupling. In addition, our results raise the possibility that the three proteins form pre-assembled complexes before connecting to the plasma membrane, providing a new insight into the formation of JMCs. In general, increasing evidence suggest that JPH2 does not only provide a structural support for the maintenance of JMCs but plays a multifaceted role in cardiomyocytes. A recent study indeed showed that, under stress conditions, the cleavage of JPH2 liberates a fragment that regulates the transcription of a spectrum of genes exerting a protective effect against pathological remodelling.^52^

A surprising result in the present study was the similar reduction in Ca_v_1.2 expression after 4 days of culture in both control myocytes and cells overexpressing JPH2, while LTCC activity was increased in the latter group. It has been demonstrated that, once internalized, voltage gated Ca^2+^ channels can be sequestered to a metabolically stable pool for at least 24 h, instead of being degraded.^53^ Similar findings have been documented for voltage gated Na^+^ channels and it was proposed that this intracellular pool of inactive channels formed a reserve for rapid incorporation to the membrane when needed.^54^ A fraction of Ca_v_1.2 might similarly persist in cultured myocytes and be recruited to the membrane in cells overexpressing JPH2.

### 4.3 Lipid rafts are involved in the modulation of LTCC activity

As expected, the number of LTCCs was significantly reduced in remodelled T-tubules following cholesterol depletion. Interestingly, disrupting lipid rafts also reduced LTCC open probability, as a high proportion of channels in the crest could not be recorded without an agonist. The LTCC accessory subunit Ca_v_β2 has been shown to increase Ca_v_1.2 open probability in rat cardiomyocytes.^55^ Our proximity ligation assays showed a significant reduction in JPH2-Ca_v_β2 interaction after cholesterol removal. We therefore suggest that the decrease in channel open probability resulted from the dissociation of Ca_v_β2 from LTCC complexes. While most Ca_v_β subunits are cytosolic, at least one splice variant of Ca_v_β2, Ca_v_β2a, binds to the membrane due to the palmitoylation of two cysteine residues.^56^ This post-translational lipid modification is involved in the targeting of many proteins to lipid rafts.^57^ It is therefore possible that Ca_v_β2 associates with lipid rafts where it interacts with Ca_v_1.2 (*Figure 7*), this interaction being impaired if lipid rafts are disrupted following cholesterol removal.

LTCC activity and open probability are also modulated by direct phosphorylation of the channel which can be regulated by various G protein-coupled receptors (GPCRs) such as β-adrenergic receptors (β-AR) (reviewed in reference 58). In fact, it was recently shown that the interaction of Ca_v_1.2 with Ca_v_β subunits is necessary for the β-AR regulation of LTCCs.^59^ It is well recognized that caveolae serve as platforms for the assembly of signalling complexes that include receptors, downstream effectors, and targets.^60^ Indeed, Ca_v_1.2 was shown to associate with Cav3 and components of the β-AR pathway within lipid rafts membrane fractions.^61^^,^^62^ We suggest that, as in caveolae, Cav3 and cholesterol provide a scaffold in T-tubules to organize signalling components for the regulation of LTCCs. Supporting this hypothesis, Cav3 was shown to play a role in the phosphorylation of Ca_v_1.2 in T-tubules, under basal condition and upon stimulation of β_2_-AR.^63^ In addition, our group previously demonstrated that the localization and signalling of β_2_-AR in T-tubules are affected by cholesterol depletion and Cav3 mutation.^64^^,^^65^

The higher proportion of inactive LTCCs in the crest compared to T-tubules following lipid raft disruption suggests that the channels are regulated differently between the two membrane domains. This agrees with our previous work which showed different membrane compartmentation of β1- and β2-AR signalling.^64^^,^^65^

Experiments using MβCD to deplete membrane cholesterol have yielded contradicting results. Our group previously showed that acute treatment of rat atrial myocytes with MβCD reduced *I*_Ca,__L_ due to a decrease in sarcolemmal LTCC activity.^40^ No effects were however observed on LTCC activity and open probability in T-tubules. This is possibly due to the short incubation time with MβCD (30 min) which might not be long enough to affect T-tubules. In fact no effect was observed on T-tubule distribution and density. In a study using rat ventricular myocytes, *I*_Ca,__L_ was not impaired by 1 h incubation with MβCD.^66^ A similar treatment, however, reduced *I*_Ca,__L_ amplitude and modified LTCCs gating properties in skeletal myotubes.^67^ The effect of MβCD on LTCCs is therefore unclear, discrepancies possibly arising from the different cell type used and the variability in experimental protocols. In the present study, cells were kept in culture for 4 days which affected basal LTCC activity as shown by an increased open probability, which was decreased after cholesterol removal. Depleting membrane cholesterol in freshly isolated cells is therefore likely to produce different results.

## 5. Conclusion

Based on our results and previous publications, we propose a model where cholesterol and Cav3 support the assembly of lipid rafts in T-tubules which provide an anchor for JPH2 to form JMCs. We hypothesize that JPH2 directly interacts with both Ca_v_1.2 and RyR to facilitate CICR and ensure a highly efficient EC-coupling. We further suggest that the rafts contain molecules that modulate LTCC activity such as accessory LTCC subunits. Finally, when cholesterol is depleted from the membrane, the dissociation of lipid rafts leads to the loss of tethering partners for JPH2, eventually resulting in the disruption of JMCs and the remodelling of T-tubules, while raft proteins such as Cav3 are relocated to the outer membrane. Further experiments should aim at identifying the lipid raft component(s) involved in the binding of JPH2 to T-tubules. The extent of the role played by Cav3 within JMCs should also be clarified, by, for instance, monitoring the effect of JPH2 overexpression on T-tubules and LTCCs in the presence of a mutant version of Cav3.

## Supplementary material

Supplementary material is available at *Cardiovascular Research* online.

## Supplementary Material

cvaa033_Supplementary_Data

## Notes

**Time for primary review: 42 days**

## Notes

Translational perspective By tethering T-tubules to the sarcoplasmic reticulum, junctophilin-2 (JPH2) supports the assembly of junctional membrane complexes (JMCs), major sites of excitation–contraction coupling in cardiomyocytes. JPH2 downregulation underlies the disruption of JMCs and T-tubules in cardiomyopathies and enhancing JPH2 expression is a promising therapeutic approach for the treatment of heart failure. Our study demonstrates a new role for JPH2 in the recruitment of calcium channels to the membrane and provides innovative insights into the formation and organization of JMCs as well as into the regulation of excitation–contraction coupling. Our results are of significant importance when considering JPH2 as a therapeutic target.
