# Supplementary material for: Junctophilin-2 tethers T-tubules and recruits functional L-type calcium channels to lipid rafts in adult cardiomyocytes
Source: Cardiovasc Res. 2020 Feb 13;117(1):149–61. doi: 10.1093/cvr/cvaa033 (PMC7797210; doi:10.1093/cvr/cvaa033)
Supplement: cvaa033_Supplementary_Data [file cvaa033_supplementary_data.zip › cvaa033-suppl_data/CVR-2019-0750R1 Supplementary Material 200130.pdf]

Supplementary Material

Junctophilin-2 tethers T-tubules and recruits functional L-type calcium channels to lipid rafts in adult cardiomyocytes

Claire Poulet[a], Jose Sanchez-Alonso[a], Pamela Swiatlowska[a], Florence Mouy[a], Carla Lucarelli[a,b], Anita Alvarez-Laviada[a], Polina Gross[c], Cesare Terracciano[a], Steven Houser[c], Julia Gorelik[a].

[a] National Heart and Lung Institute
Imperial College London
London, United Kingdom

[b] Department of Cardiac Surgery
School of Medicine
University of Verona
Verona, Italy

[c] Cardiovascular Research Center
Lewis Katz School of Medicine Temple University,
Philadelphia, PA, USA

Corresponding author:
Julia Gorelik
ICTEM
Du Cane Road,
London W12 0NN
United Kingdom
j.gorelik@imperial.ac.uk>
+44 (0)20 7594 2736

Detailed Methods

Cardiomyocyte isolation and culture

Our isolation and culture methods are based on protocols that have been optimized for the maintenance of cardiomyocytes in culture (reviewed in [1]). Sprague-Dawley rats (150–250 g) were anesthetized with 5% isoflurane-95% O2 and then killed by cervical dislocation. Hearts were fast extracted and placed in Tyrode solution containing in (mmol/L): 140 NaCl, 6 KCl, 1 $MgCl_2$, 1 $CaCl_2$, 10 glucose and 10 HEPES, adjusted to pH 7.4 with 2 mmol/L NaOH. Using aortic cannulation with the Langendorff setting, the hearts were perfused with Tyrode solution for 5 min, then with low Ca^{2+} solution containing in (mmol/L): 120 NaCl, 5.4 KCl, 5 $MgSO_4$, 5 sodium pyruvate, 20 glucose, 20 taurine, 10 HEPES, 5 nitrilotriacetic acid, and 0.04 $CaCl_2$, adjusted to pH 6.96 with 2 mmol/L NaOH for 5 min, and finally for 10 min with enzyme solution containing in (mmol/L): 120 NaCl, 5.4 KCl, 5 $MgSO_4$, 5 sodium pyruvate, 20 glucose, 20 taurine, 10 HEPES, and 0.2 $CaCl_2$, pH 7.4 with collagenase (1 mg/ml; Worthington) and hyaluronidase (0.6 mg/ml). The ventricles were cut into small pieces, resuspended in enzyme solution containing only collagenase (1 mg/ml; Worthington) and shaken in a water bath at 37°C for 20 minutes. The cell suspension was then filtered through a 200-µm nylon mesh and washed twice before plating.

Feline cardiomyocyte isolation and transduction

Felines were anesethetized using 50 mg/kg sodium pentobarbital and hearts were excised and washed in with Krebs Henseleit Buffer (KHB) (12.5 mmol/L glucose, 5.4mmol/L KCl, 1 mmol/L lactic acid, 1.2 mmol/L MgSO4, 130 mmol/L NaCl, 1.2 mmol/L NaH2PO4, 25 mmol/L NaHCO3, and 2 mmol/L Na-pyruvate, and aerated with 95% oxygen and 5% CO2, pH 7.35-7.4, and warmed to 37ºC). Following aortic cannulation, retrograde perfusion on a Langendorff apparatus was performed with KHB, followed by digestion buffer (KHB with 180 U/mL collagenase and 50 µmol/L CaCl2). The ventricles were isolated and minced. Cardiomyocytes were filtered, equilibrated in KHB supplemented with 200 µmol/L CaCl2 and 1% bovine serum albumin (BSA) at room temperature, washed with Medium 199 (Sigma) plus penicillin-streptomycin-glutamine (PSG) (Gibco), and plated on laminin (BD Bioscience)-coated culture plates. After 2 hours, medium was changed to Medium 199 supplemented with PSG, 5 mmol/L taurine (Sigma), 5 mmol/L creatinine (Sigma), and 2 mmol/L carnitine (Sigma).

Myocytes were infected with adenovirus (Ad) expressing JPH2-HA tag for 12-hours at a multiplicity of infection of 100 and then changed to long-term culture media. During the experimental period, culture media was changed once per day. Infection efficiency was determined 36-48 hours after infection by HA immunofluorescence staining and was typically assessed to be 85%.

For generation of human JPH2-HA adenoviral construct, the pShuttle vector (Bio Basic, Amherst, NY, USA) was modified to contain a N-terminal HA peptide sequence downstream of the multiple cloning site. Human JPH2 cDNA was PCR amplified to contain a 5' BamHI sequence and to alter the stop codon to a 3' BamHI site. Adenovirus was made as made as previously described[2,3] (Vector Biolabs, Malvern, PA, USA).

Real time RT-PCR

For rat myocytes, total RNA from cells was isolated using the peqGOLD Total RNA kit (PeqLab) according to the manufacturer's instructions. RNA was cleared off genomic DNA by DNase digestion (DNase I digest kit, PeqLab). Gene expression was analysed using the QuantiTect SYBR Green RT-PCR Kit (Qiagen) and polymerase (RNA) II (DNA directed) polypeptide A as housekeeping gene (Rn_Polr2a_1_SG QuantiTect Primer Assay, Qiagen). Primers were designed to target jph2 (F 5'-AGTACGAGGGCACTTGGAATAAC-3', R 5'-GTGAATTGGCCTTGGTAGGTC-3'), cav3 (F 5'-ACTGCAAGGAGATAGACTTGGTG-3', R 5'-GACGGTGAAAGTGGTGTAGCTC-3') and cacna1c (F 5'-CAGCATTGTTGAATGGAAACC-3', R 5'-GTGGCGTTGGAGTCATCTTC-3').

For feline myocytes, RNA was extracted from with TRIzol Reagent. The RNA was cleaned using the Quick-RNA™ MiniPrep (Zymo Research) clean-up protocol. Reverse transcription (RT) was performed using the SuperScript III first strand synthesis system for RT-PCR (Invitrogen) and

oligo-dt primers according to the manufacturer's instructions. Real-time PCR was performed using the Quantifast Sybrgreen PCR kit (Qiagen). Data was normalized to GAPDH. Primers were designed for feline JPH2 (F 5'-CCCCAACACTGTCCTCATCT-3', R 5'-CCAGAGTCCAGTCCTCCTTG-3'); human JPH2 (F 5'-ATACTGGAGCCAGGGCAAAC-3', R 5'-TGAACTGGCCTTGGTACGTC-3') and feline GAPDH (F 5'-ACAGTCAAGGCTGAGAACGG-3', R 5'-TACTTCTCGTGGTTCACGCC-3').

Immunostaining & T-tubule staining

For immunostaining myocytes on coverslips were fixed with 4% formaldehyde for 10 min, permeabilized using 0.5% Triton X-100 for 15 min and blocked with 10% FCS in PBS for 30 min at room temperature. Incubation with primary antibodies was done in blocking buffer for 2-3h at room temperature or overnight at 4°C. Subsequent incubation with secondary antibodies AlexaFluor488 or AlexaFluor546 (Invitrogen) was performed in blocking buffer for 1h at room temperature.

For T-tubule staining, live myocytes on coverslips were washed once with PBS, incubated with 10 mmol/L Di-8-ANEPPS (Molecular Probes, Eugene, OR, USA) for 1 min and then washed twice with PBS. Images were taken with a Zeiss LSM-780 inverted confocal microscope.

Super-resolution scanning patch clamp

After a 3D image was generated by SICM using a sharp pipette (~ 100 nm inner diameter, 100MΩ resistance) the pipette was moved to a cell-free area and clipped by a controlled impact on the bottom of the dish to increase the size of the pipette tip (ranging from 281 to 711 nm inner diameter), thereby decreasing the pipette resistance (ranging from 15 to 38 MΩ). After moving the pipette back to a position of choice on the 3D scan, it was lowered to touch the cell and gentle suction was applied to form a gigaseal. Cell-attached patch-clamp recordings of single LTCCs were then performed with an Axopatch 200A amplifier (Axon Instruments, Foster City, CA, USA) and pClamp software version 10 (Axon Instruments). Experiments were done at room temperature with the following bath solution (in mmol/L): 120 K-gluconate, 25 KCl, 2 $MgCl_2$, 1 $CaCl_2$, 2 EGTA, 10 Glucose, 10 HEPES, pH 7.4 with NaOH. The pipette solution consisted of (mmol/L): 90 $BaCl_2$, 10 HEPES, 10 Sucrose, pH 7.4 with TEA-OH. High K^+ concentration was used in the bath to zero the resting membrane potential and allow a precise control of the voltage. Data were corrected for a liquid junction potential of -16.7 mV. Channel density per μm^2 was calculated by normalizing the total number of channels recorded to the total area of pipette openings. Channel open probability was calculated using Clampfit 10.7 software (Axon Instruments) and averaged from 10-20 traces per cell.

Whole cell patch-clamp

Whole cell Ca^{2+} currents were measured using an Axopatch-1D amplifier (Molecular devices LLC, Sunnyvale, CA, USA) and pClamp software version 8 (Molecular Devices LLC). Experiments were performed at RT (20-23 °C). Extracellular recording solution was of the following composition (in mmol/L): 120 TEA-chloride, 10 CsCl, 10 Glucose, 10 HEPES, 1 $MgCl_2$, 2 $CaCl_2$, pH 7.4 with TEAOH. Patch pipettes with a mean resistance of 3-4 MΩ were filled with an intracellular solution containing (in mmol/L): 100 Cs-methanesulfonate, 40 CsCl, 10 HEPES, 5 EGTA, 5 ATP-Mg salt, 1 $MgCl_2$, , pH 7.3 with CsOH. Depolarizing pulses were applied from a holding potential -40 mV to test potentials ranging from –45 mV to +60 mV of 200 ms duration. The bath was connected to the ground via an Ag–AgCl pellet. Series resistance and cell capacitance were compensated to 80 %.

Western Blot

To extract total proteins, cells were put on ice and washed once with cold PBS before lysis with RIPA buffer (Sigma) supplemented with 10µM protease inhibitor (Sigma). After using a cell scraper to ensure that all cells were detached and lysed, the cell suspension was collected, sonicated for 1 min 4°C, mixed thoroughly for 1 min, shaken on ice for 15 min and finally centrifuged 15 min at 14000 rcf and 4°C. The supernatant was transferred to a fresh tube. To quantify protein concentration BCA assay was performed using (Pierce BCA Protein Assay kit). Samples were resolved on a SDS-PAGE electrophoresis gel. Trans-Blot Turbo Transfer System was used to transfer proteins to the PVDF membranes, which then were blocked for 1 hour in 5% milk (Sigma) and incubated overnight, 4°C with

primary antibodies; Cav3 (1:1000,mouse, BD Biosciences), JPH2 (1:1000,mouse, Santa Cruz Biotechnology), GAPDH (1:1000, rabbit, Santa Cruz Biotechnology). Membranes were washed in TBST buffer and incubated 1 hour with secondary antibodies (anti-mouse, anti-rabbit HRP-linked Cell signalling, 1:1000), washed in TBST again, incubated with ECL substrate(ECL BioRad Substrate Clarity Western), and imaged. Image analysis were performed using ImageJ software.

Supplementary Figures

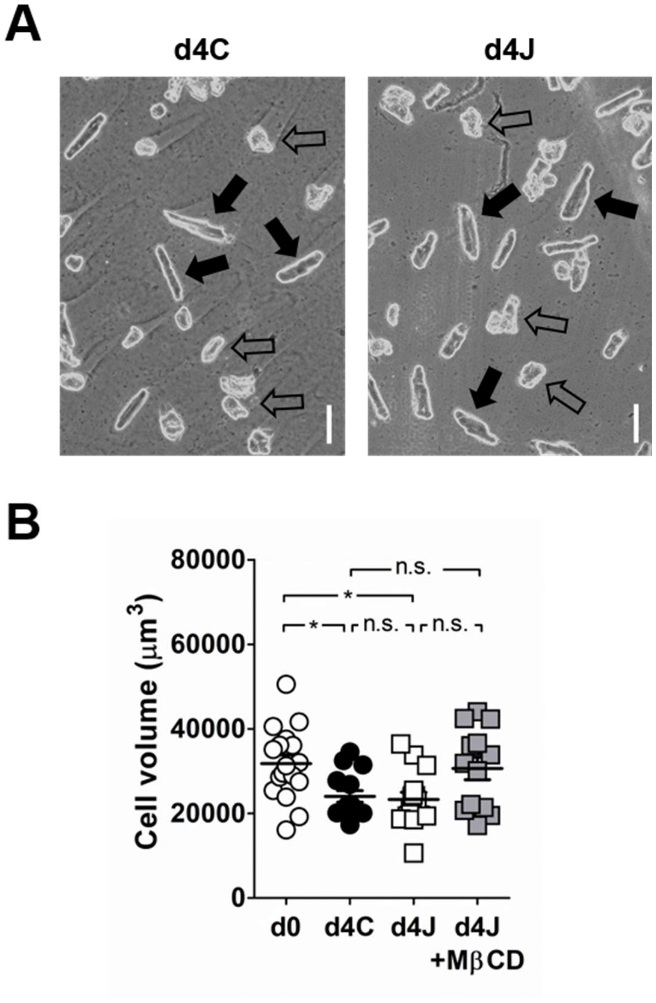

Supplementary Figure S1

A) Morphology of control myocytes (d4C) and myocytes overexpressing JPH2 (d4J) after 4 day of culture. Examples of cells that kept an elongated shape are shown by filled arrows. Examples of cells that collapsed are shown by empty arrows. Scale bar: 100 µm. B) Cell volume was measured in freshly isolated cardiomyocytes (d0, n = 17) and after 4 days of culture in control cells (d4C, n = 15) or in cells overexpressing JPH2 in basal conditions (d4J, n = 15) or chronically treated with 100 µmol/L MβCD (d4J + MβCD, n = 13). After 4 days of culture, cell volume was only measured in cells that kept an elongated shape. *$p < 0.05$, one-way ANOVA followed by Tukey's multiple comparison test.

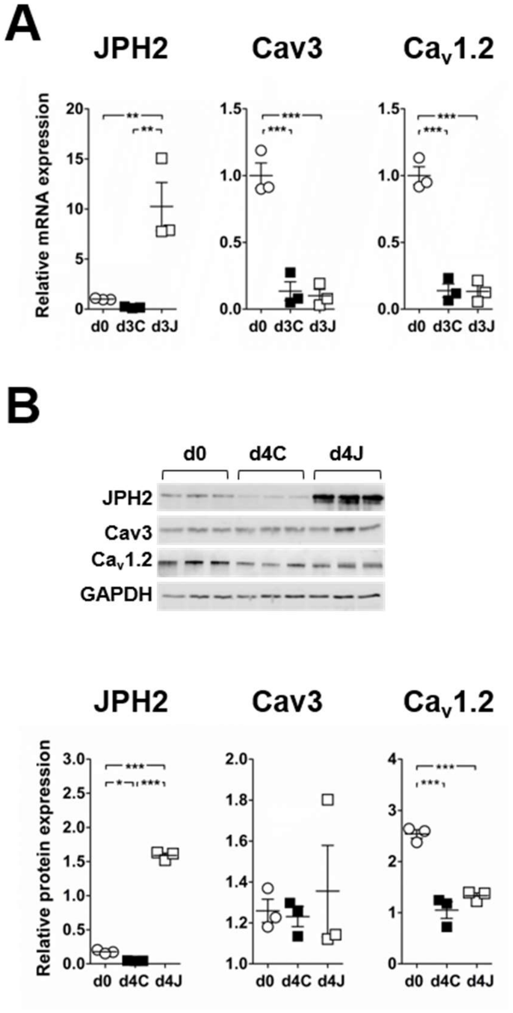

Supplementary Figure S2

A) Gene expression in freshly isolated cardiomyocytes (d0) and after 3 days of culture in control cells (d3C) or in cells overexpressing JPH2 (d3J). B) Protein expression in freshly isolated cardiomyocytes (d0) and after 4 days of culture in control cells (d4C) or in cells overexpressing JPH2 (d4J). The number of isolations is given in the columns. *p < 0.05, **p < 0.01, ***p < 0.0001, one-way ANOVA followed by Tukey's multiple comparison test.

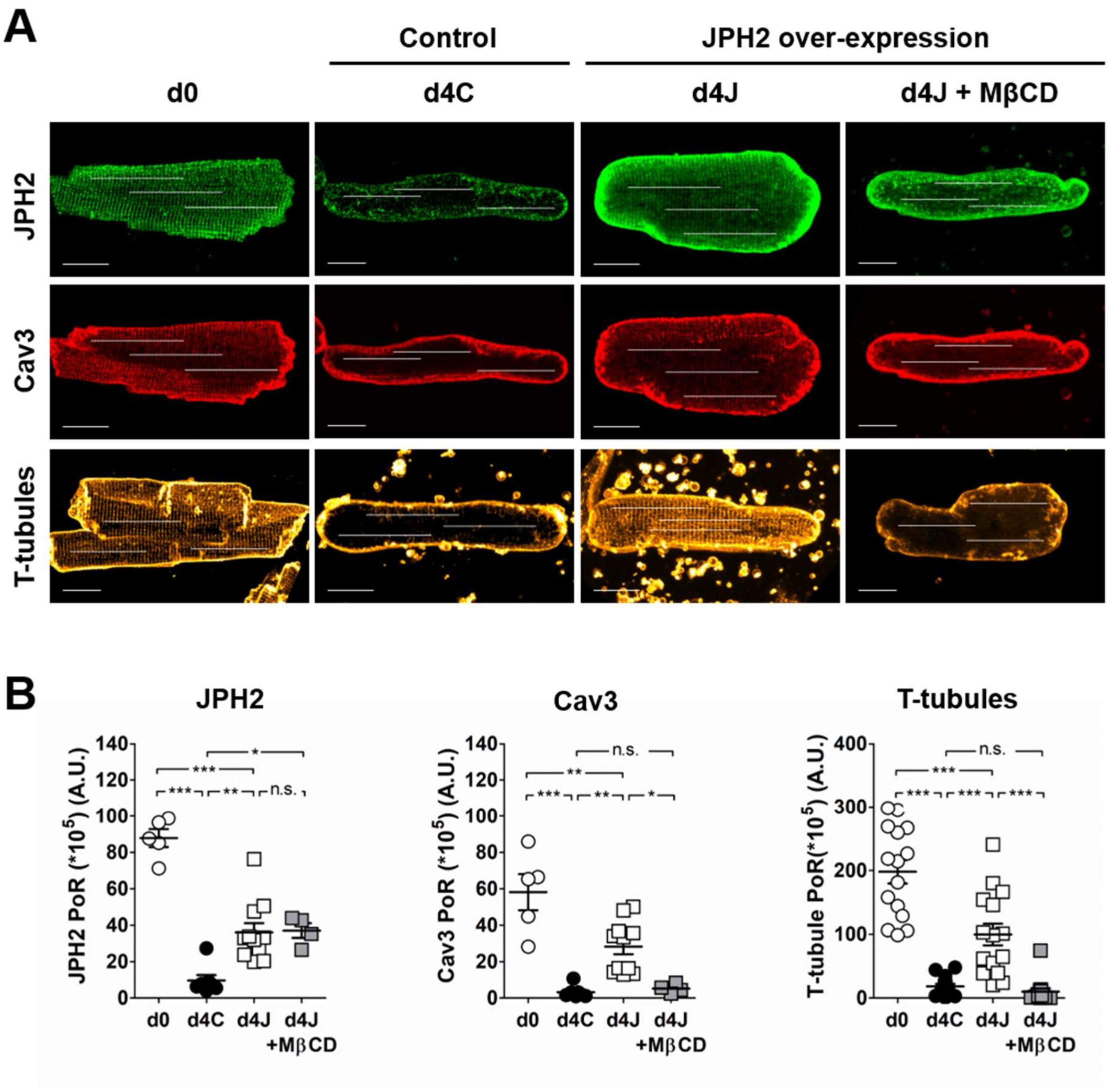

Supplementary Figure S3

Maximum intensity projections were generated from z-stacks covering an averaged thickness of 9.28 ± 0.03 µm. A) Examples of representative Z-projections showing the distribution of JPH2, Cav3 and T-tubules in freshly isolated cardiomyocytes (d0) and after 4 days of culture in control cells (d4C) or in cells overexpressing JPH2 in basal conditions (d4J) or chronically treated with 100 µmol/L MβCD (d4J + MβCD). Scale bar : 20 µm. Distribution regularity was measured in 3 different areas of each cell (40 µm-long white lines) and is shown in B). The dots represent the number of myocytes. For JPH2 and Cav3 stainings: n = 5 (d0); n = 7 (d4C); n = 11 (d4J); n = 13 (d4J + MβCD). For T-tubule staining: n = 15 for d0, d4C and d4J; n 0 13 for d4J + MβCD. *p < 0.05, **p < 0.01, ***p < 0.0001, one-way ANOVA followed by Tukey's multiple comparison test.

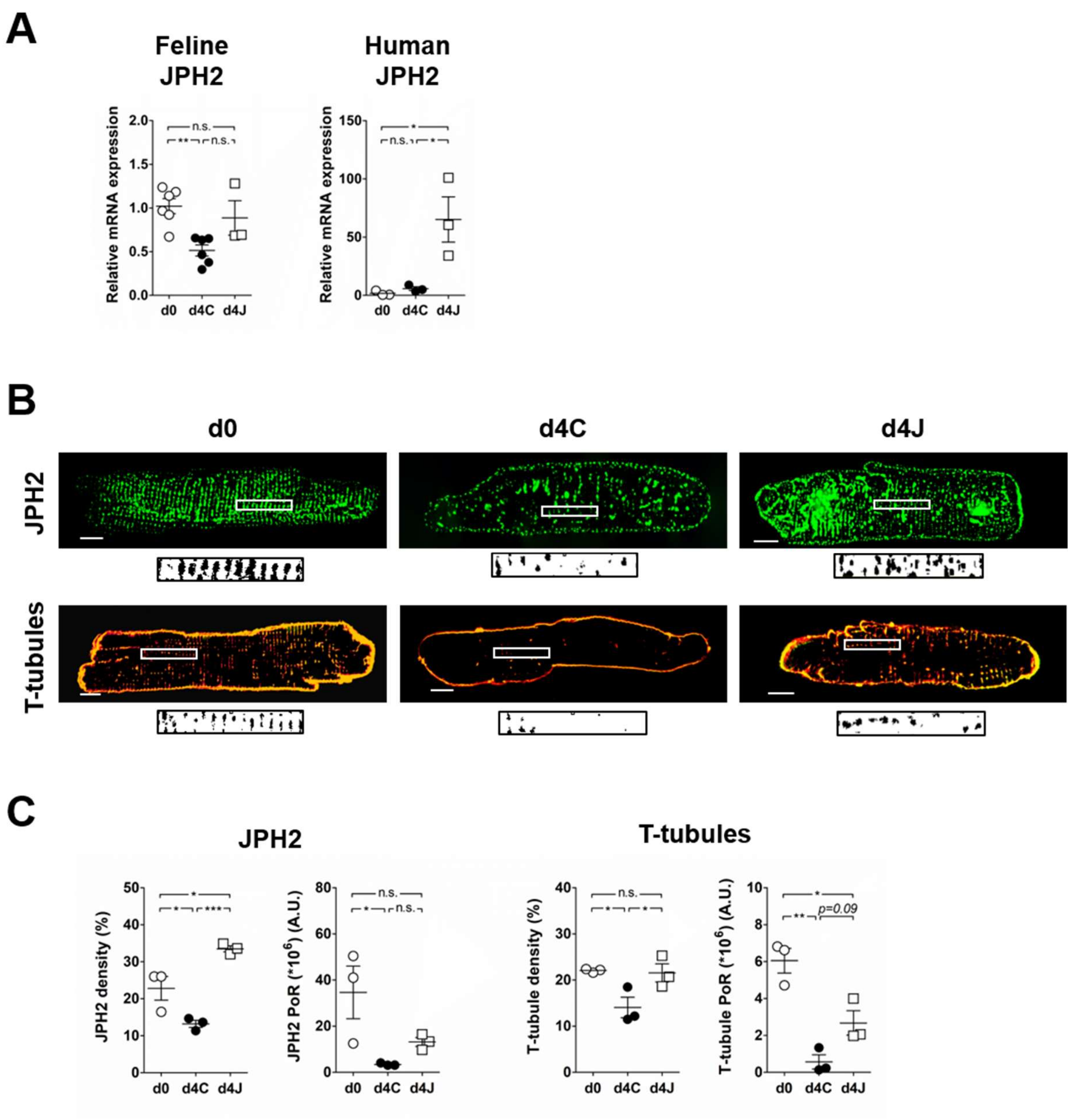

Supplementary Figure S4

A) Gene expression in freshly isolated adult feline ventricular myocytes (d0) and after 4 days of culture in control cells (d4C) or in cells overexpressing JPH2 (d4J). Levels for endogenous feline JPH2 and exogenous human JPH2 were measured. $^{**}p < 0.01$, $^{***}p < 0.0001$, one-way ANOVA followed by Tukey's multiple comparison test. B) Distribution of JPH2 and T-tubules in freshly isolated adult feline ventricular myocytes (d0) and after 4 days of culture in control myocytes (d4C) or in myocytes overexpressing JPH2 (d4J). Scale bar: 20 µm. The entire cell was selected to calculate staining density. An area of 40x5µm was selected in each cell (white rectangle) as shown under each picture to calculate the power of regularity. Represented scale is 10µm. C) Average values were calculated from 9-10 cells stained for JPH2 and 4-6 cells stained for Di-8-ANEPPS per isolation. The number of isolations is given in the columns. $^{*}p < 0.05$, $^{**}p < 0.01$, $^{***}p < 0.0001$, one-way ANOVA followed by Tukey's multiple comparison test.

Supplementary Movies

Examples of z-stacks showing JPH2 (green) and Cav3 (red) or T-tubules (orange). The display range of the images was left unaltered. A gaussian blur filter (sigma 1.5) was applied before saving them as AVI files. Scale bar: 10 µm.

Supplementary References

1. Louch WE, Sheehan KA, Wolska BM. Methods in cardiomyocyte isolation, culture, and gene transfer. J. Mol. Cell. Cardiol. 2011.
2. Wu X, Eder P, Chang B, Molkentin JD. TRPC channels are necessary mediators of pathologic cardiac hypertrophy. *Proc Natl Acad Sci* 2010;**107**:7000–7005.
3. Xu J, Gong NL, Bodi I, Aronow BJ, Backx PH, Molkentin JD. Myocyte enhancer factors 2A and 2C induce dilated cardiomyopathy in transgenic mice. *J Biol Chem* 2006;**281**:9152–9162.
